# Optimizing the precursor of sulfur source for hydrothermal synthesis of high performance CdS for photocatalytic hydrogen production†

**DOI:** 10.1039/c8ra00250a

**Published:** 2018-03-22

**Authors:** Hui Li, Lihua Liu, Ziqun Wang, Xiuzhen Zheng, Sugang Meng, Shifu Chen, Xianliang Fu

**Affiliations:** College of Chemistry and Material Science, Huaibei Normal University Huaibei Anhui 235000 China zyxyz0804@163.com fuxiliang@gmail.com

## Abstract

Although the CdS photocatalyst has been extensively investigated, a rational hydrothermal synthesis route is still required to prepare highly active CdS for H_2_ evolution reaction (HER). To optimize the precursor of the sulfur source, three prevalent organic sulfur sources of thiourea (TA), thioacetamide (TAA) and l-cysteine (l-Cys) were used for hydrothermal synthesis of CdS. Their effects on the crystallographic structure, morphology, optical property, band structure, and photocatalytic HER performance of the products were then investigated systematically. The results indicated that hexagonal branched dendritic structure CdS (S-TA) could be produced in TA solution and showed the highest HER activity due to the branched 1D structure, the smallest interfacial electron transfer resistance and the most negative conduction band bottom (*E*_cb_). Whereas in TAA, spherical CdS (S-TAA) with a mixed phase of hexagonal and cubic was obtained. The mixed phase structure and the more positive *E*_cb_ of S-TAA lead to a considerably lower HER activity than that of S-TA. Poorly crystallized hexagonal CdS nanoparticles (S-Cys) were prepared in l-Cys and showed the lowest HER performance as its *E*_cb_ is very near to H^+^ reduction potential. Thus, compared to T-AA and l-Cys, TA is a more suitable sulfur source for hydrothermal preparation of highly active CdS for HER.

## Abstract 2

The precursor of sulfur source for hydrothermal synthesis of high performance CdS for photocatalytic hydrogen production has been optimized.

## Graphical abstract

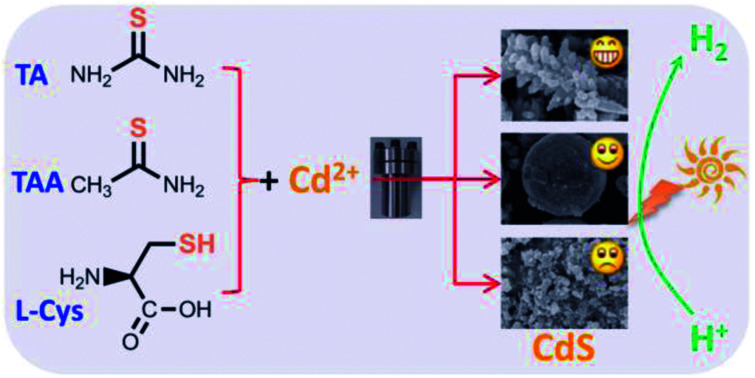

## 1. Introduction

As a clean and high density energy carrier, H_2_ has been considered as an ideal alternative to fossil fuel to solve the increasing energy demands and the serious environmental issues caused by extensive use of fossil fuels.^1^ However, at present H_2_ is mainly produced by steam reforming of nonrenewable fossil fuel or electrolysis of water. These high-energy consumption processes are unsustainable, environmentally unfriendly, and cost-expensive. Solar-driven photocatalytic H_2_ evolution from a renewable resource like water^2^ or biomass derivatives^3^ is highly desirable because it provides a sustainable, clean, and low-cost route for H_2_ production. The photocatalytic H_2_ evolution reaction (HER) first involves the generation of photoinduced electron (e^−^) and hole (h^+^) pairs in the bulk of a photocatalyst under irradiation, then the transfer of charge carriers from the photocatalyst bulk to its surface, where the HER is finally induced by e^−^.^4^ The key challenge for photocatalytic HER is to develop a highly efficient visible light photocatalyst which essentially determines the harvesting of light and the conversion efficiency of solar to H_2_.

Since the first report of photoelectrochemical water splitting on TiO_2_ by Honda and Fujishima in 1972,^5^ various photocatalysts have been developed for photocatalytic HER.^2,6^ CdS is one of the most attractive and widely investigated prototypes due to its moderate band gap energy (*ca.* 2.4 eV) and the deep negative conduction band edge position, which corresponds well with the solar spectrum and the redox potential of H_2_O/H_2_. To pursue a highly efficient HER performance, many efforts have been devoted to manipulate the intrinsic structure features of CdS, including the crystalline phase,^2,7,8^ particle size, and morphology,^9–14^ to optimize its electronic and optical properties, while other works^7,15–22^ mainly focus on the modification of CdS with a highly effective or economical cocatalyst. However, it should be noted that these manipulations and modifications are essentially determined by the synthesis methods as the structure, grain size, morphology, and component of CdS-based photocatalyst are changed with the preparation conditions.^23–27^ Thus, to pursue a high HER performance, the preparation route and the reaction conditions of CdS need to be optimized.

Generally, CdS can be synthesized by several methods including thermal evaporation, chemical vapor deposition, precipitation followed by thermal treatment, solvothermal, and hydrothermal routes.^13,28–34^ Among them, the hydrothermal process provides a facile one-pot and environmental route to synthesize nanostructure CdS at a low temperature and it has a great potential for the large-scale preparation of CdS. Besides, the structure, morphology and component of CdS also can be readily tuned by this route through the change of the reaction conditions, such as the composition of the solution, the treatment temperature and the reaction time.^13,31,34–36^ One of the key factors to affect the property of CdS is the precursor of sulfur. Generally, for the preparation of nanostructure CdS, organic sulfur sources such as thiourea (TA), thioacetamide (TAA) and l-cysteine (l-Cys) are more popular than inorganic one like Na_2_S.^35,37–43^ It can be ascribed to the fact that, compared to the rigid inorganic sulfur sources, organic sulfur sources have distinct advantages in flexibility, structure variety and shape diversity, which can not only serve as a precursor of S^2−^, but also as a complexing agent for Cd^2+^ due to the presence of –NH_2_ and –COOH ligand groups to control the nucleation and the growth of CdS.^43–46^ Furthermore, under hydrothermal treatment, S^2−^ can be released gradually from these organic sulfur sources, which provides favorable conditions for the nucleation and the growth of CdS. Consequently, the structure and morphology of CdS can be manipulated by using different sulfur source. However, the effect of organic sulfur source on these aspects, as well as the photocatalytic activity of CdS, has been seldom investigated, especially for hydrothermal synthesis of CdS.^35,47,48^

Herein, three prevalent sulfur sources including TA, TAA, and l-Cys were used for hydrothermal synthesis of CdS in this work. The effects of these sulfur sources on the properties of the resulted CdS were then investigated systematically, including the crystal structure, morphology, optical property, band structure, and photocatalytic performance for HER. The purpose of this paper is to provide a reasonable reference for hydrothermal synthesis of highly active CdS photocatalyst for HER.

## 2. Experimental

### 2.1. Preparation of photocatalysts

Cd(NO_3_)_2_·4H_2_O, TA, TAA, and l-Cys were analytical grade and used as received from Aladdin Chemical Reagent Co. Three CdS samples were prepared by hydrothermal methods with the commonly used TA, TAA, and l-Cys as the sulfur sources, respectively. The corresponding products were denoted as S-TA, S-TAA, and S-Cys. Taken the preparation of S-TA as an example, 10 mmol TA was first dissolved in 40 mL H_2_O and labelled as Solution A. Then, 3 mmol Cd(NO_3_)_2_·4H_2_O was dissolved in another 40 mL H_2_O as Solution B. Subsequently, the Solution B was added dropwise to the Solution A under vigorous stirring. The mixture was then transferred to a 100 mL Teflon-lined stainless autoclave and kept at 180 °C for 24 h. After cooling to room temperature, the resulted yellow sediment was collected by centrifugation and rinsed thoroughly with deionized water and ethanol alternately. The final product was dried in a vacuum oven at 60 °C for 10 h. The preparation of S-TAA and S-Cys is similar to that of S-TA except that the sulfur precursor was changed to 10 mmol TAA or l-Cys.

### 2.2. Characterization

The phase structure of the CdS samples was measured by X-ray powder diffraction (XRD) on a Bruker D8 Advance X-ray diffractometer using Ni-filtered Cu Kα radiation (*λ* = 1.5406 Å). UV-visible diffuse reflection spectra (UV-vis DRS) were recorded on a TU-1950 Vis-NIR spectrophotometer (TU-1950, Persee) with BaSO_4_ as a reference. Field emission scanning electron microscopy (SEM) images of the samples were observed by a Hitachi SU8000 scanning electron microscope. High-resolution/transmission electron microscopy (HR/TEM) images were performed on a JEOL JEM-2100 URP electron microscope operated at an acceleration voltage of 200 kV. X-ray photoelectron spectroscopy (XPS) analysis was conducted on an ESCALAB 250 photoelectron spectrometer (Thermo Fisher Scientific) at 3.0 × 10^−10^ mbar by using Al Kα X-ray beam (1486.6 eV). All binding energies were corrected to the C 1s peak of the surface adventitious carbon at 284.6 eV. The Fourier transform infrared spectrometry (FTIR) spectra of the samples were measured by a Thermo Nicolet Nexus 6700 FTIR spectrophotometer by using KBr pellets. Multipoint Brunauer–Emmett–Teller (BET) specific surface areas were determined by N_2_ adsorption isotherms on a Micromeritics ASAP 2020 surface area analyser. Steady photoluminescence (PL) emission spectra of CdS samples were recorded on a JASCO FP-6500 type fluorescence spectrophotometer excited by 418 nm light at room temperature.

### 2.3. Electrochemical measurements

The electrochemical impedance spectra (EIS) and the photocurrents of the CdS samples were measured on a CHI 660E electrochemical workstation (Chenhua, Shanghai) in 0.2 M Na_2_SO_4_ aqueous solution. A standard three-electrode cell was used for the measurements with the prepared CdS, an Ag/AgCl (3 M KCl), and a Pt plate as the working, reference, and counter electrode, respectively. The working electrode was prepared by drop-coating CdS/ethanol suspension on FTO glass (0.6 × 0.6 cm^2^). A 300 W Xe lamp equipped with a UV cut filter (*λ* > 420 nm) was used as the excitation source. The irradiation was chopped manually every 20 s during the photocurrent measurements. The Mott–Schottky (M–S) analysis was performed based on the EIS measurements with a potential ranged from −1.0 to 0.2 V (*vs.* Ag/AgCl) at an AC frequency of 1.5 kHz. The flat-band potentials of the CdS then can be estimated from the intersection of the M–S plots. The measured potentials (*vs.* Ag/AgCl) were converted to NHE scale by *E*(NHE) = *E*(Ag/AgCl) + 0.197.

### 2.4. Photocatalytic HER test

The photocatalytic HER performances of the prepared CdS were conducted on a commercial LabSolar II (Perfect Light Co.) reaction system. 50 mg CdS, 90 mL water, 10 mL lactic acid (as the electron donor), and 1 mL H_2_PtCl_6_ solution (corresponding to 1 wt% Pt loading amount, used as cocatalyst for HER) were added to a top-irradiation reaction cell. The system was then evacuated by a mechanical pump. Before irradiation, the suspension was stirred in the dark for 30 min to reach an adsorption–desorption equilibrium. A 300 W Xe lamp (PLS-SEX 300, Perfect Light Co., Beijing) equipped with a UV cut filter (*λ* > 420 nm) was used as a visible light source. The average intensity of the irradiation for the activation of CdS is *ca.* 1.1 mW cm^−2^ (420–570 nm, the spectrum range for the activation of CdS) and the irradiation area is *ca.* 28 cm^2^ (Fig. S1, see ESI†). Pt will be deposited on CdS after turning on the lamp and then serves as a cocatalyst for HER. The solution temperature was controlled at 10 °C during the test by a water-circulating unit. During the reaction, the generated H_2_ was measured by an online gas chromatograph (GC7900, TianMei, Shanghai).

## 3. Results and discussion

**Fig. 1:**
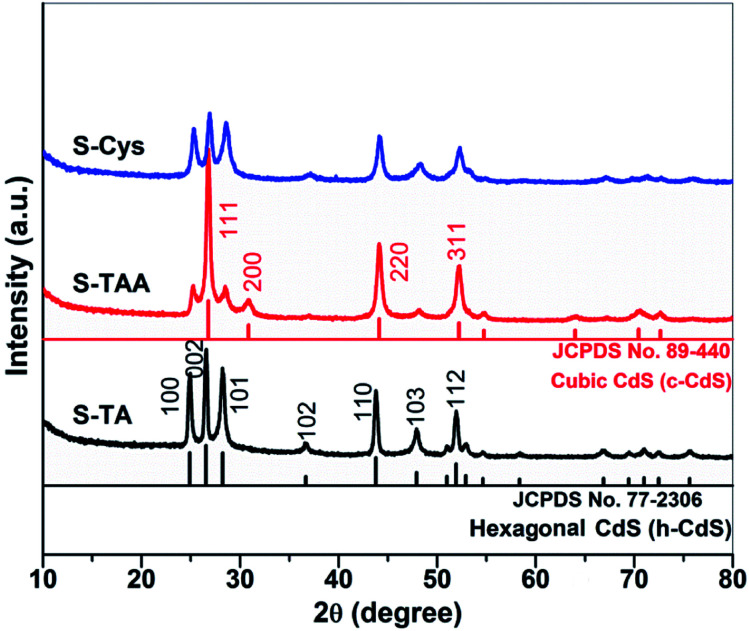
XRD patterns of the CdS samples prepared in different sulfur source solution.

Fig. 1 shows the XRD patterns of the CdS synthesized in the different sulfur source solutions. It indicates that the samples prepared in TA and l-Cys solutions (S-TA and S-Cys) have a wurtzite structure and the diffraction patterns can be indexed to hexagonal CdS (h-CdS) with lattice constants of *a* = 4.14 and *c* = 6.71 Å, which agree well with the values of JCPDS No. 77-2306. The diffraction peaks at 2*θ* = 24.9, 26.5, 28.2, 36.7, 43.8, 47.9, and 51.8 degree correspond to the (100), (002), (101), (102), (110), (103), and (112) facets of h-CdS. Compared to S-TA, the low and broad diffraction peaks of S-Cys suggest that both the crystallinity and the particle size of S-Cys were suppressed with l-Cys as the sulfur source. The average particle size of S-Cys is estimated to be only *ca.* 32 nm according to the Scherrer equation.^49^ However, when TAA was used as the sulfur source, the resulted sample (S-TAA) was a mixture of h-CdS and metastable cubic CdS (c-CdS)^30^ (JCPDS No. 89-440), which is consistent with Dai's work.^50^ The peaks located at 2*θ* = 26.9, 31.0, 44.3, and 52.3 degree can be indexed to the (111), (200), (220) and (311) planes of c-CdS. Apparently, the diffraction peaks of c-CdS are more pronounced than that of h-CdS. A semi-quantitative analysis method has been used to calculate the fraction of h- and c-CdS in S-TAA based on the reference intensity ratio (RIR) values available from the JCPDS cards. The result indicates that S-TAA contains *ca.* 45% hexagonal and 55% cubic CdS. The XRD result demonstrates that the phase structure of the prepared CdS is closely related to the sulfur source. TA and l-Cys favor the formation of h-CdS, while TAA benefits the reservation of metastable c-CdS.

**Fig. 2:**
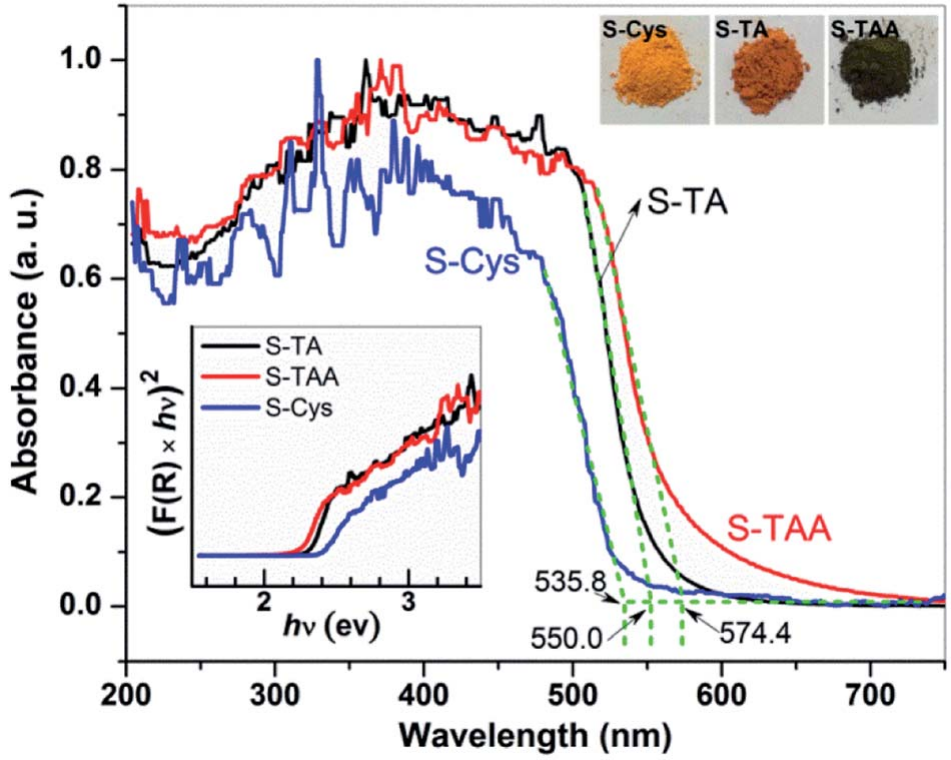
UV-vis DRS of the CdS samples prepared in different sulfur source solutions.

The optical property of the prepared CdS was investigated by UV-vis DRS. As shown in Fig. 2, a steep visible absorption with a different edge can be observed in the samples. It can be ascribed to the intrinsic band gap transition of CdS. According to the intercept of the tangent of the absorption curve, the threshold of the absorption locates at *ca.* 555.0, 574.4, and 535.8 nm for S-TA, S-TAA, and S-Cys, respectively. S-TAA and S-TA have a wider visible light absorption than S-Cys. The difference in the absorption property of the CdS samples is also reflected in the change of their colors (see the inset of Fig. 2), from yellow (S-Cys) to brown (S-TA) and finally to dark green (S-TAA). The green color of S-TAA implies that the sample may be contaminated by amorphous C formed during the hydrothermal process as similar color has been reported on carbon spheres supported CdS.^51^

Tauc's plots^52^ was used to estimate the band gap energy (*E*_g_) of the CdS samples: *K*(*hv*−*E*_g_)^1/*n*^ = *F*(*R*)*hv*, where *F*(*R*) is the absorption coefficient, *hv* is photon energy, *K* is a constant, and *n* = 2 as CdS is a direct band gap material.^30,32,38^ As shown in the inset of Fig. 2, the *E*_g_ of S-TAA, S-TA, and S-Cys are determined to be 2.2, 2.3, and 2.4 eV, respectively. The *E*_g_ of the mixture phase S-TAA is lower than that of the hexagonal S-TA and S-Cys. This result agrees well with the reported works.^12,45^ Besides, the differences in particle size and the morphology of the prepared CdS may also account for their different absorption features.^53,54^

**Fig. 3:**
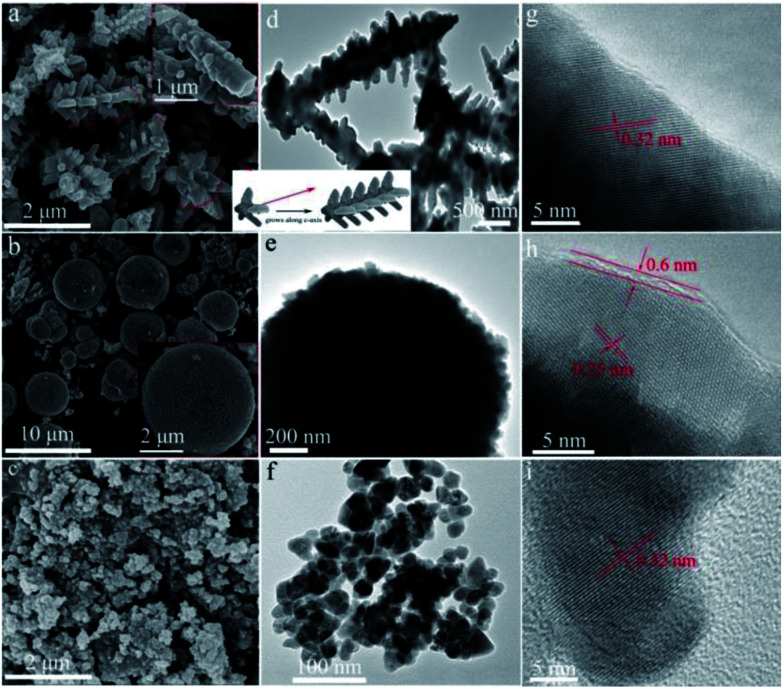
(a–c) SEM, (d–f) TEM and (g–i) HRTEM images of the prepared CdS samples: (a, d, g) S-TA, (b, e, h) S-TAA, and (c, f, i) S-Cys.

The prepared CdS samples were characterized by SEM and TEM to reveal their morphologies. As shown in Fig. 3a and d, the sample prepared in TA solution exhibits a dendritic-like architecture. Some branches grown along the trunk in five directions can be observed. The diameter and length of the trunk are *ca.* 0.3 and 2.5 μm, respectively, while the corresponding parameters of the branches are *ca.* 0.1 and 0.2 μm. As indicated by the arrows in Fig. 3a, the branches in each direction parallel to each other. It suggests that a pentagon-like CdS block may firstly form in the early reaction stage. With hydrothermal time increased, the block then grows longitudinally along the *c*-axis (as illustrated in the inset of Fig. 3a) to result in the final branched 1D structures as the growth rate of hexagonal CdS in *c*-axis is usually faster than other directions.^36^ As displayed in Fig. 3b and e, when TAA was used as the sulfur source, spherical CdS was obtained with a scale of 3–6 μm. Enlarged SEM (inset of Fig. 3b) and the TEM images indicate that the CdS spheres are formed by the aggregation of many primary particles. For the preparation performed in l-Cys solution, the resulted S-Cys (Fig. 3c–f) is mainly composed of nanoparticles with average size *ca.* 30 nm. The size is in agreement with the result calculated by Scherrer formula in the XRD pattern (Fig. 1). Fig. 3g–I show the HRTEM images of the prepared CdS samples. Two sets of lattice fringes can be observed with interplanar spacing of 0.32 and 0.25 nm, which can be ascribed to the (101) and (102) planes of hexagonal CdS. A layer of impurity with a thickness of *ca.* 0.6 nm can be observed on S-TAA (Fig. 3h). Considering that S-TAA presents a same green color with C spheres supported CdS,^51^ the layer should be amorphous C which is formed from the carbonization of TAA during the hydrothermal treatment. However, similar amorphous layer on S-TA and S-Cys is inconspicuous.

**Fig. 4:**
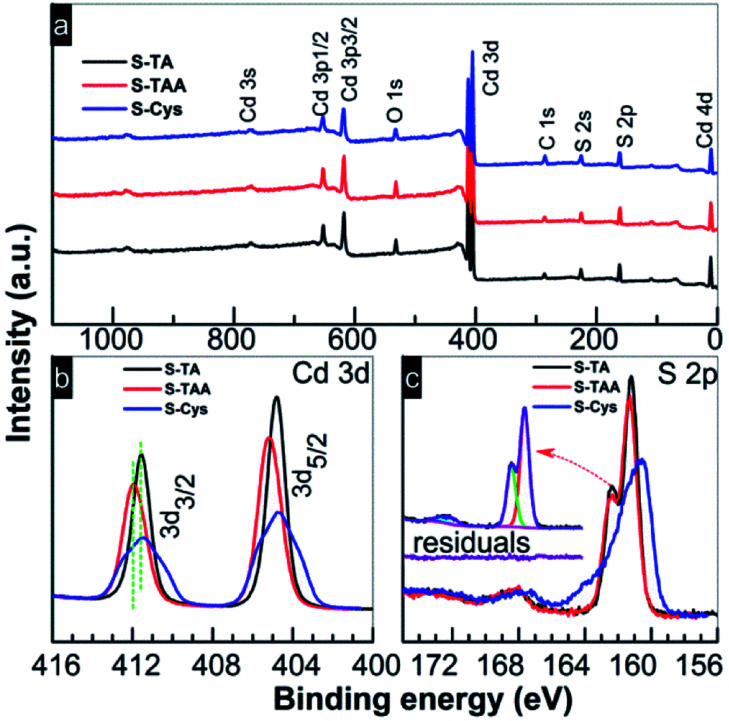
(a) Survey and high resolution XPS spectra of (b) Cd 3d and (c) S 2p of the CdS prepared in different sulfur source solutions.

XPS was used to analyse the elemental composition and the chemical states of the prepared CdS. As shown in the survey spectra (Fig. 4a), in addition to the adventitious C and the adsorbed O, the samples are composed of Cd and S. The characteristic N 1s (at *ca.* 397 eV) signal cannot be observed suggesting that the sulfur sources have been removed completely by the washing process. The atomic ratios of Cd to S are summarized in Table 1 and the results are close to the nominal composition of CdS. Two peaks centered at the binding energy (BE) of 404.5 (Cd 3d_5/2_) and 411.2 (Cd 3d_3/2_) eV can be found in the high resolution spectrum of Cd 3d (Fig. 4b). The BE values and the splitting energy of 6.7 eV are consistent with the reported results of Cd^2+^ in CdS.^55,56^ Compared to the hexagonal CdS samples (S-TA and S-Cys), the mixed phase S-TAA shows a positive shift of 0.4 eV in the binding energy of Cd 3d. The spectrum of S 2p (Fig. 4c) indicates that the peak can be deconvoluted into two peaks (see the inset in Fig. 4c, taken S-TA sample as an example), one located at *ca.* 161.0 and the other at *ca.* 162.1 eV with an energy difference of 1.1 eV. The peaks can be assigned to the characteristic S 2p_3/2_ and S 2p_1/2_ peaks of S^2−^ in CdS.^56^ Another doublet peak of S 2p can be further perceived on these CdS samples with S 2p_3/2_ and S 2p_1/2_ at 168.0 and 169.1 eV, respectively, although the signals are weak. These peaks can be ascribed to SO_4_^2−^ or SO_3_^2−^ species.^57,58^ It suggests that an oxidation of the CdS surface occurred during the hydrothermal treatment, which results in the fact that the actual atomic ratio of S is slightly less than the theoretical value as shown in Table 1. Our previous work^59^ indicated that the formation of these oxides can be avoided by a solvothermal preparation method with ethanediamine as a solvent. Thus, under the hydrothermal conditions, the sulfation of CdS should be facilitated by H_2_O or the dissolved O_2_. Compared to S-TA and S-TAA, the XPS peaks of Cd 3d and S 2p in S-Cys become broader. The small grain size of S-Cys is responsible for the broadening of the peaks due to the increasing inhomogeneity of Cd and S atoms in small particles.

**Table 1:** Summarize the preparation, characterization, and photocatalytic HER activity of the prepared CdS samples

Samples	Sulfur source	Phase^a^	*E*_g_ b eV	BET m^2^ g^−1^	*E*_cb_ c eV	*E*_vb_	*r*_H_2__ d	At%^e^	
								Cd	S
S-TA	TA	h-	2.3	2.2	−0.59	1.71	1.60	53.8	46.2
S-TAA	TAA	h&c-	2.2	4.7	−0.48	1.72	0.43	53.0	47.0
S-Cys	l-Cys	h-	2.4	20.2	−0.40	2.00	0.10	55.8	44.2

a h-, hexagonal, c-, cubic phase CdS.

b Estimated by Tauc plots.

c Estimated by *E*_fb_.

d mmol h^−1^ g^−1^.

e Atomic ratio, measured by XPS.

**Fig. 5:**
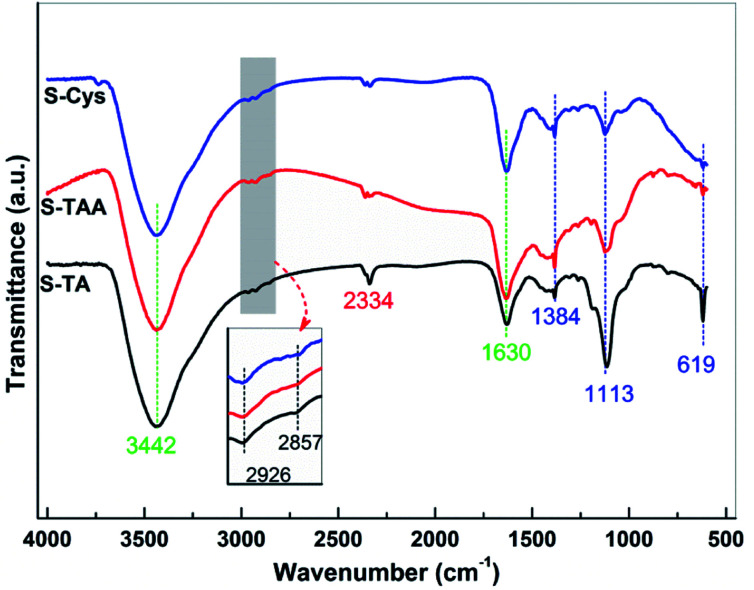
FTIR spectra of the prepared CdS samples.

FTIR spectroscopy was used to further investigate the surface structure of the CdS samples. The spectra are depicted in Fig. 5. The broad band centred at 3442 cm^−1^ can be assigned to the O–H stretching vibration of the adsorbed H_2_O from the atmosphere, while the bands at 1630 cm^−1^ can be attributed to the corresponding O–H bending vibration.^60,61^ Besides H_2_O, the asymmetric stretching vibration of adsorbed CO_2_ also can be observed at 2334 cm^−1^.^61,62^ The bands located at 1384, 1113, and 619 cm^−1^ can be ascribed to the typical characteristic IR absorption of Cd–S bond,^60,61,63^ confirming the formation of CdS. The sulfation of CdS is further confirmed by the FTIR results as the bands at 2926 and 2857 cm^−1^ can be observed, which can be assigned to the asymmetric and symmetric stretching vibration of Cd–O, respectively.^57^ The bending vibration of N–H and the stretching vibration of C–N (around 1100 and 1500 cm^−1^, respectively^64,65^) have not been observed, eliminating the possibility of the sulfur precursors adsorbing on the samples surface. Thus, it can deduce that there is no significant difference in the surface structure of the synthesized CdS samples.

**Fig. 6:**
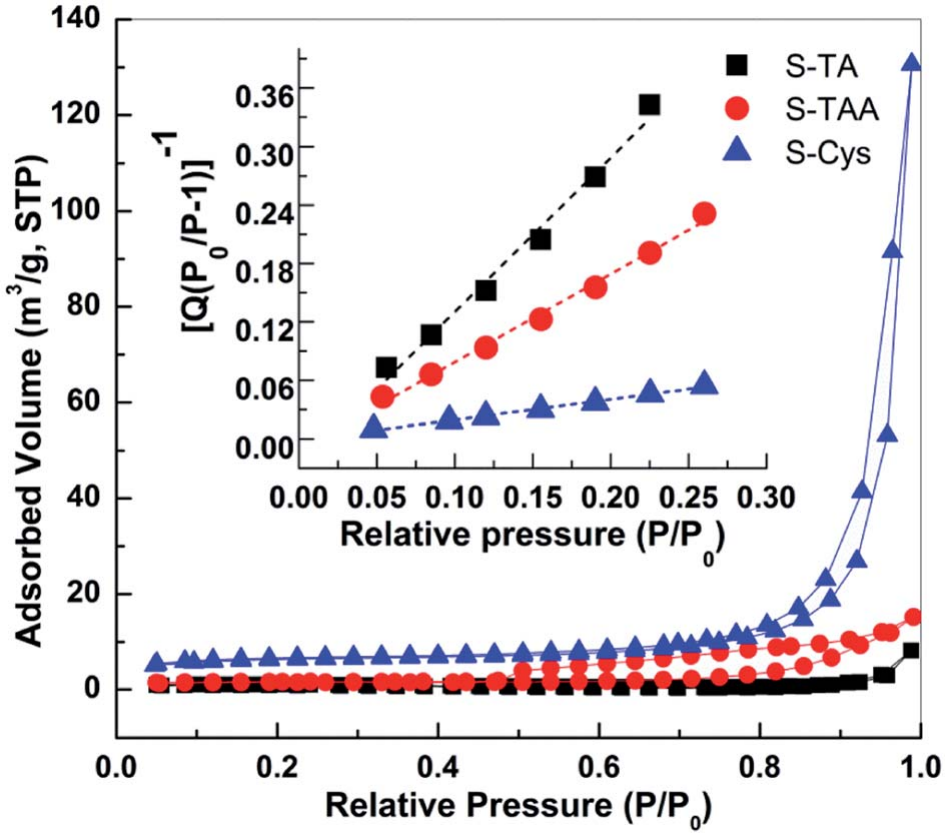
N_2_ adsorption–desorption isotherms of prepared CdS samples. Insert shows the BET transform plots of 1/*Q*[(*P*_0_/*P*)−1] *versus P*/*P*_0_.

The BET surface areas (*S*_BET_) of the CdS samples were measured by N_2_ adsorption at 77 K. Fig. 6 shows the N_2_ adsorption–desorption isotherms of the prepared CdS. The curves of S-Cys and S-TAA are of a type V isotherm with a H3 type of hysteretic loop according to the IUPAC classification, suggesting a disordered mesoporous structure formed by the aggregation of small particles. A similar type of isotherm curve with no apparent hysteretic loop is observed on S-TA. The BET surface areas (*S*_BET_) of the CdS samples then can be calculated from the transform plot of 1/*Q*[(*P*_0_/*P*)−1] *versus P*/*P*_0_ (see the insert of Fig. 6), where *Q* is the quantity of the adsorbed N_2_ (cm^3^ g^−1^, STP). The results indicate that the surface area decreases in order S-Cys (20.2) > S-TAA (4.7) > S-TA (2.2 m^2^ g^−1^).

**Fig. 7:**
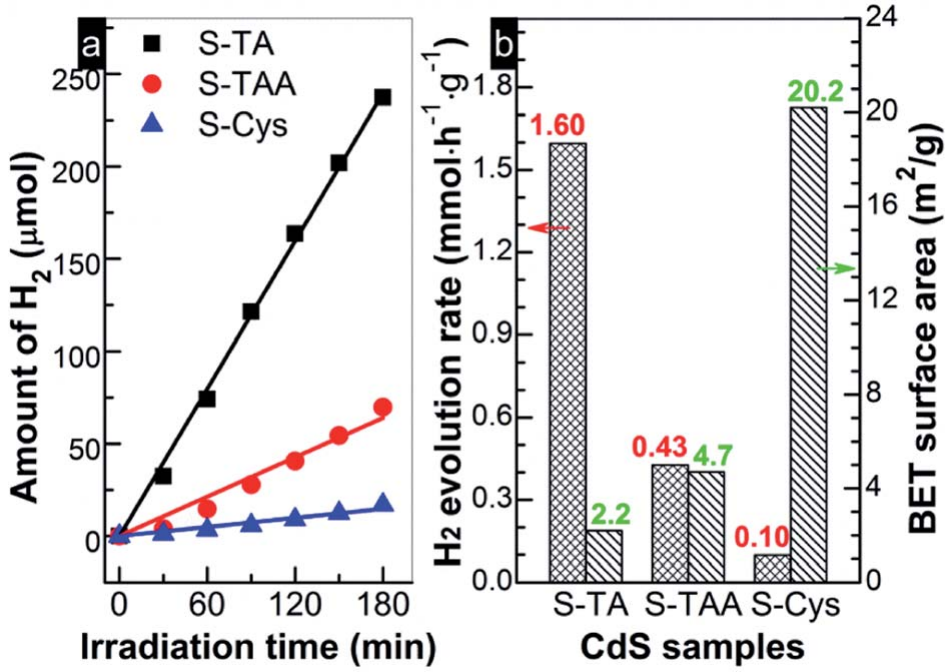
(a) Time course of H_2_ evolution and (b) H_2_ evolution rates and BET surface area obtained on the prepared CdS samples.

Fig. 7 shows the photocatalytic HER activity of the prepared CdS under visible light irradiation (>420 nm). A linear increase of H_2_ with irradiation time can be found for all tests. The evolution of H_2_ amount on the CdS samples decreases in order S-TA > S-TAA > S-Cys. S-Cys showed the lowest activity and only 16.9 μmol H_2_ was produced after irradiation for 3 h. Controlled tests indicated that no H_2_ was detected in the absence of CdS or irradiation, suggesting that the evolution of H_2_ on the CdS samples was triggered by a photocatalytic process. The *r*_H_2__ of these samples can be measured according to the fitting lines' slopes and the results are displayed in Fig. 7. Obviously, S-TA shows the highest activity for H_2_ production than other samples and the value of *r*_H_2__ (1.6 mmol h^−1^ g^−1^) is *ca.* 4 and 16 times higher than that of S-TAA (0.43) and S-Cys (0.10 mmol h^−1^ g^−1^), respectively. An association analysis was then conducted between the HER performances and the *S*_BET_. As shown in Fig. 7b, there is no rational correlation between them. Although S-TA has the smallest area, it shows the highest activity, while S-Cys with the largest surface area shows the lowest activity. Consistent with Bao's work,^45^*S*_BET_ is not a key factor to restrict the HER performance of CdS. The different HER activities of CdS should be attributed to other factors, such as their intrinsic crystallographic and the band structures because the separation of photoinduced charge carriers and the proceeding of HER will be determined by these structures.

**Fig. 8:**
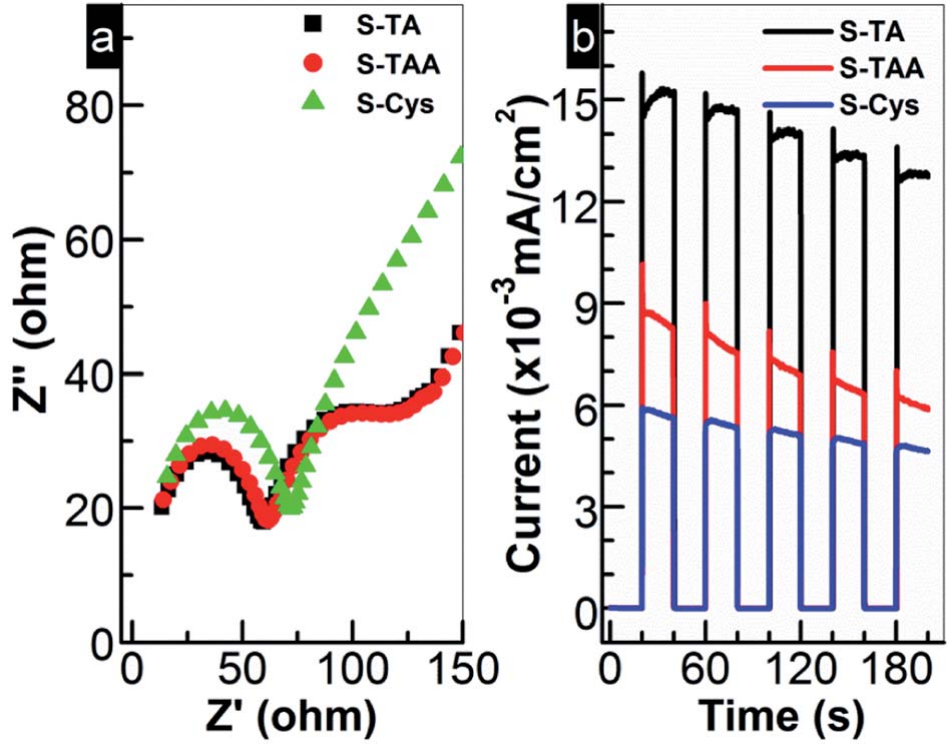
(a) ESI Nyquist plots and (b) transient photocurrent responses of the prepared CdS samples.

Generally, the photocatalytic HER is controlled kinetically by the amount of charge carriers (e^−^ and h^+^) on the photocatalyst surface and thermodynamically by the band edge potentials of the photocatalyst. To interpret the HER performance of the prepared CdS, the EIS analysis was first carried out to investigate the migration of e^−^ and h^+^. As shown in Fig. 8a, the charge transfer resistance from CdS to the surface can be estimated by the semi-arc radius in the low frequency region. Small radius suggests a low interfacial e^−^ transport resistance. A high separation efficiency of photoinduced e^−^ and h^+^ then can be expected. For the prepared CdS, the circular radius decreases in order of S-Cys > S-TAA ≈ S-TA. The radius of S-TAA is slightly larger than that of S-TA and both are substantially smaller than that of S-Cys. It suggests that S-TA and S-TAA possess a good e^−^ conductivity, while S-Cys has the largest e^−^ transport resistance. The low crystallinity and small particle size of S-Cys account for the largest resistance as more defects and grain boundaries will be introduced. Thus, it can speculate that S-TA and S-TAA will show higher efficiency for the separation of e^−^ and h^+^ than that of S-Cys. To confirm this assumption, the photocurrents of the CdS samples were measured. Indeed, as shown in Fig. 8b, the photocurrent density drops in order of S-TA > S-TAA > S-Cys. Surprisingly, although the interfacial e^−^ transport resistance of S-TAA is comparable to that of S-TA, the photocurrent density of S-TAA is inferior to S-TA. Nevertheless, the photo-electrochemical results are roughly consistent with the HER activity.

**Fig. 9:**
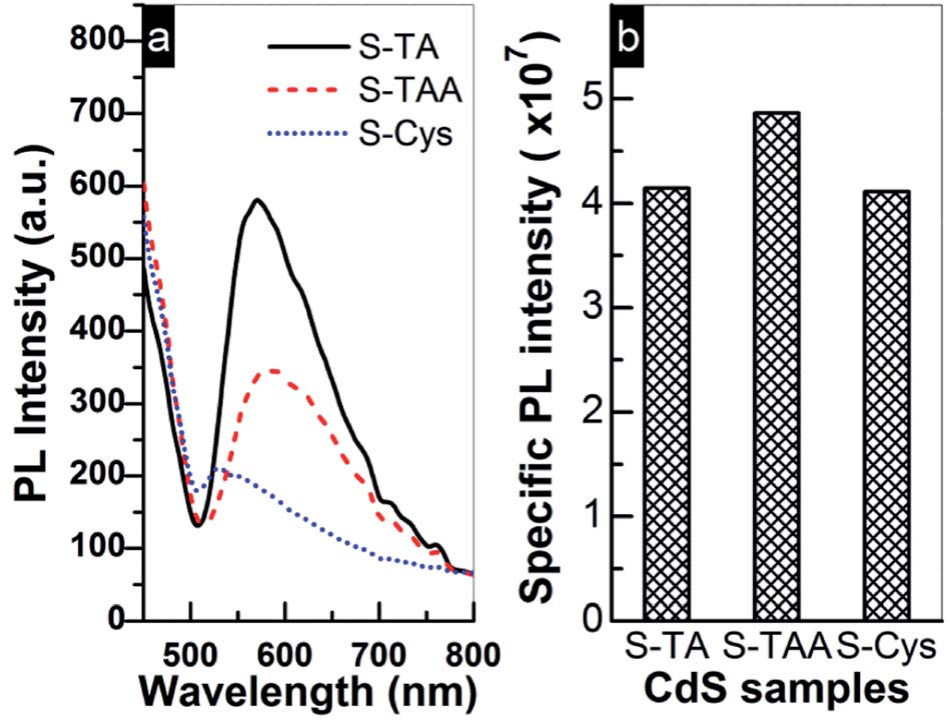
(a) PL emission spectra of prepared CdS samples excited by 418 nm light at room temperature and (b) comparison of the photocurrent specific PL intensity.

The above results indicate that S-TAA has the widest absorption range (Fig. 2) and low charge transfer resistance. Therefore, the inferior photocurrent response of S-TAA should be ascribed to other causes rather than to the light absorption or the transfer of charge carriers. It is more likely to be caused by the mixed phase structure of S-TAA, which facilitates the recombination of interfacial charge carriers. Similar assignment has been reported. To confirm this conjecture, the steady PL spectra of the CdS samples were recorded because the technique can provide useful information about the recombination of photoinduced charge carriers on a photocatalyst surface. As demonstrated in Fig. 9a, a broad emission band ranging from 500 to 800 nm with a different intensity can be observed for all CdS samples. The band should be produced by the superposition of the band-edge emission (around 520 nm) and some shallow and deep trap-state-related emissions of CdS.^25,66^

Usually a high PL intensity indicates a quick recombination rate of charge carriers and consequently leads to a low photocatalytic performance. But for a series of photocatalysts, this conclusion must be based on the same premise that the available number of e^−^ and h^+^ for the recombination process is comparable on these photocatalysts. This is because the PL emission intensity is not only determined by a recombination rate of e^−^ and h^+^, but also by their concentrations on the surface. A weak PL emission may be caused by the fact that the amount of e^−^ and h^+^ involved in the recombination is small rather than a low recombination rate. Thus, the density of photoinduced charge carriers must be taken into account when using steady PL to estimate the recombination efficiency of charge carriers. Based on this consideration, the photocurrent specific PL intensity of the prepared CdS was compared in Fig. 9b, which is calculated by dividing the PL intensity of CdS with the corresponding photocurrent intensity (shown in Fig. 8b and used as a reference to estimate the amount of surface charge carriers). Apparently, the specific PL intensity of the mixed phase S-TAA is more intensive than that of S-TA and S-Cys, while the specific PL intensity of the hexagonal phase S-TA and S-Cys is comparable to each other. The results indicate that the mixed phase S-TAA does have higher recombination efficiency of e^−^ and h^+^ pairs than that of the hexagonal phase S-TA and S-Cys. This finding also suggests that the lowest PL intensity of S-Cys shown in Fig. 9a should be caused by a low concentration of photoinduced charge carriers due to its high charge transfer resistance.

**Fig. 10:**
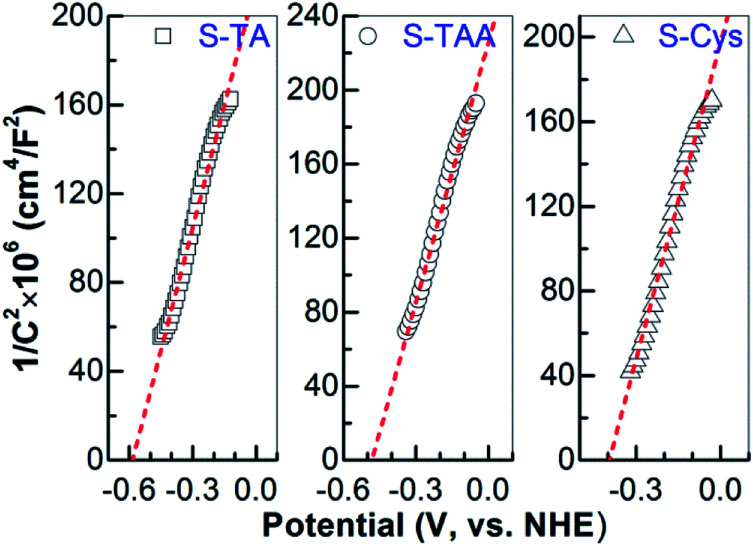
Mott–Schottky plots of the CdS prepared in different sulfur source solutions.

The band edge potential of CdS is another key factor to determine the HER activity, which thermodynamically decides whether the HER can be occurred. The more negative of the conduction band (CB) edge, the easier the evolution of H_2_. Mott–Schottky (MS) plot analysis of the prepared CdS was then performed to investigate their band structures. The positive slopes of the plots (Fig. 10) suggest a n-type semiconductor characteristic of the CdS samples. Thus, the CB edge potential (*E*_cb_) of CdS is approximately equal to the flat band potential (*E*_fb_)^67^ which can be measured by the interception of the tangent to the *X* axis in the MS plot. The *E*_cb_ values are estimated to be −0.59, −0.48 and −0.40 V (*vs.* NHE, pH 7) for S-Ta, S-TAA and S-Cys, respectively. Obviously, the photogenerated e^−^ on S-TA has the strongest reduction capability, followed by S-TAA, while on S-Cys, it shows the lowest reduction power for H_2_ evolution. In fact, the CB edge of S-Cys is very near to H^+^ reduction potential of −0.41 V (*vs.* NHE, pH 7).^68^ That means the HER will be hard to proceed on S-Cys. This is the critical reason why S-Cys shows the lowest HER performance in Fig. 6. However, S-TA and S-TAA did not encounter such problem due to the more negative *E*_cb_ and they consequently exhibit substantial HER activities.

The above results indicate that the crystallographic structure, morphology, optical property, and band structure of the hydrothermally prepared CdS are significantly affected by the used sulfur source. The combination of these factors results in a different HER performance. This is mainly reflected in the following three aspects. First, in terms of the crystallographic structure, some works^7,12,27^ demonstrated that the HER activity of CdS benefited from the hexagonal rather than the cubic structure CdS which commonly shows a low crystallinity and contains a lot of defects.^29^ The separation of photoinduced charge carriers will be suppressed by these defects as they can serve as recombination sites.^2,7,8^ This has been confirmed by the observation that the mixture phase S-TAA shows a lower photocurrent density and stronger PL emission than that of S-TA, even though they possess almost the same interfacial e^−^ transport resistance (Fig. 8a). Besides, the crystalline defects formed at the interface of the two phases are also responsible for the high recombination of e^−^ and h^+^ in S-TAA.^45^ Second, the HER performance is further affected by CdS morphology. It has been extensively reported that^27,28,35^ 1D nanostructure CdS show a much higher activity than other morphology samples because the separation of charge carriers can be promoted by this high aspect ratio structure through increasing the delocalization of e^−^ on the 1D structure. Thus, compared to spherical S-TAA and polygonal S-Cys, branched 1D structure S-TA has an inherent advantage for HER in terms of morphology. Third, the HER is controlled thermodynamically by CdS band structure. The CB minimum determines whether the HER can be proceeded, which requires that the CB edge potential should be more negative than *E*(H^+^/H_2_) (−0.41 V, *vs.* NHE, pH 7). For specific CdS sample, the band structure is changed with its particle size, crystal structure and morphology,^27,29,35,53^ which essentially depend on the preparation method. In this work, MS plot analysis indicates that CdS prepared in the different sulfur source solutions show different CB minimum. But only the CB minimums of S-TA and S-TAA are lower than H^+^ reduction potential and consequently exhibit substantial HER activity.

Thus, based on these considerations, the highest HER activity of S-TA can be ascribed to the superposition of several favourable factors, including the hexagonal crystalline structure, the branched 1D morphology, and the most negative CB edge. Compared to S-TA, the inferior activity of S-TAA is mainly caused by the mixed phase structure and the more positive CB minimum, while the lowest HER activity of S-Cys is severely restricted by its band structure and the high interfacial e^−^ transport resistance. Therefore, among the commonly used TA, TAA, and l-Cys, TA is a more suitable sulfur source for hydrothermal preparation of highly active CdS than TAA and l-Cys.

## 4. Conclusions

The present work clearly indicates that when TA, TAA, and l-Cys are used as the sulfur sources for hydrothermal preparation of CdS, the resulted samples have apparent differences in crystal structure, morphology, optical property and band structure, which then lead to different HER performances. Specifically, hexagonal branched dendritic structure CdS (*i.e.* S-TA) can be prepared in TA solution. The sample has the smallest interfacial electron transfer resistance and the most negative conduction band bottom, and consequently shows the highest HER activity. As for the synthesis performed in TAA solution, spherical CdS with a mixed phase of hexagonal and cubic is obtained (*i.e.* S-TAA). The recombination of photoinduced charge carriers was facilitated by the mixed phase structure, which, together with the more positive CB minimum, leads to a considerably lower HER performance of S-TAA than that of S-TA, although they show almost the same interfacial electron transfer resistance. Low crystallized hexagonal CdS nanoparticles (*i.e.* S-Cys) with no specific morphology were prepared with l-Cys as the sulfur source. S-Cys shows the largest interfacial electron transfer resistance and its CB minimum is very near to H^+^ reduction potential. These disadvantages result in the lowest HER activity of S-Cys. Thus, compared to T-AA and l-Cys, TA is a more suitable sulfur source for hydrothermal preparation of highly active CdS for HER.

## Conflicts of interest

There are no conflicts to declare.

## Supplementary Material

RA-008-C8RA00250A-s001
