# Supplementary material for: Optimizing the precursor of sulfur source for hydrothermal synthesis of high performance CdS for photocatalytic hydrogen production
Source: RSC Adv. 2018 Mar 22;8(21):11489–97. doi: 10.1039/c8ra00250a (PMC9079128; doi:10.1039/c8ra00250a)
Supplement: RA-008-C8RA00250A-s001 [file RA-008-C8RA00250A-s001.pdf]

Electronic Supplementary Material (ESI) for RSC Advances.
This journal is © The Royal Society of Chemistry 2018

Optimizing the precursor of sulfur source for hydrothermal synthesis of high performance CdS for photocatalytic hydrogen production

Hui Li[a], Lihua Liu[a], Ziqun Wang[a], Xiuzhen Zheng[a*], Sugang Meng[a], Shifu Chen[a], Xianliang Fu[a*]

[a] College of Chemistry and Material Science, Huaibei Normal University, Huaibei, Anhui, 235000, China.

Email: X. Zheng, zyxyz0804@163.com; X. Fu, fuxiliang@gmail.com

Fig. S1 The emission spectrum of the light source.(measured by SpectriLight ILT 950 spectroradiometer).

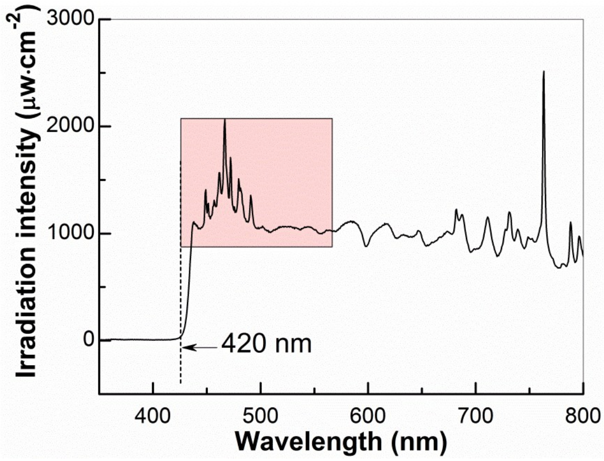

The average intensity of the irradiation for the activation of CdS is ca. 1.1 mW·cm^{-2} (420-570 nm, the spectrum range for the activation of CdS) and the irradiation area is ca. 28 cm^2.
